# BMP-Functionalised Coatings to Promote Osteogenesis for Orthopaedic Implants

**DOI:** 10.3390/ijms150610150

**Published:** 2014-06-06

**Authors:** Jianfeng Wang, Jing Guo, Jingsong Liu, Limin Wei, Gang Wu

**Affiliations:** 1 School and Hospital of Stomatology, Wenzhou Medical University, Wenzhou 325027, China; E-Mails: jwang525@gmail.com (J.W.); dentliu@aliyun.com (J.L.); 2 Department of Oral Cell Biology, Academic Centre for Dentistry Amsterdam (ACTA), Research Institute MOVE, VU University Amsterdam and University of Amsterdam, Amsterdam 1081LA, The Netherlands; E-Mail: j.guo@acta.nl; 3 Department of Oral Implantology and Prosthetic Dentistry, Academic Centre for Dentistry Amsterdam (ACTA), Research Institute MOVE, VU University Amsterdam and University of Amsterdam, Amsterdam 1081LA, The Netherlands

**Keywords:** bone morphogenetic protein, coating, orthopaedic implant, osteogenesis

## Abstract

The loss of bone integrity can significantly compromise the aesthetics and mobility of patients and can be treated using orthopaedic implants. Over the past decades; various orthopaedic implants; such as allografts; xenografts and synthetic materials; have been developed and widely used in clinical practice. However; most of these materials lack intrinsic osteoinductivity and thus cannot induce bone formation. Consequently; osteoinductive functionalisation of orthopaedic implants is needed to promote local osteogenesis and implant osteointegration. For this purpose; bone morphogenetic protein (BMP)-functionalised coatings have proven to be a simple and effective strategy. In this review; we summarise the current knowledge and recent advances regardingBMP-functionalised coatings for orthopaedic implants.

## 1. Introduction

The loss of bone integrity, which can result from congenital non-union, trauma, inflammation or osteosarcoma resection, may significantly compromise the aesthetics and mobility of patients. The osseous restoration of bone defects, particularly critical-sized defects, remains a challenge in the fields of orthopaedics, maxillofacial surgery and dental implantology [1,2]. As the “gold-standard” bone-defect-filling material, autologous bone grafts are highly osteoconductive, osteoinductive and osteogenic. However, the use of autologous bone grafts is limited by their intrinsic disadvantages, e.g., limited quantity [3] and donor site morbidity [4,5]. In many cases, an orthopaedic implant is needed to treat the loss of bone integrity. An orthopaedic implant can be defined as a medical device designed to replace a missing joint or bone or to support a damaged bone [6]. To provide viable treatment options for different bone diseases, a variety of orthopaedic implants, such as allografts, xenografts and synthetic materials, have been developed and are widely used in clinical practice. The chemical composition of orthopaedic implants can be inorganic (e.g., calcium phosphate), organic (e.g., naturally derived or synthetic polymers) or hybrid. Several novel technologies have been developed to fabricate advanced orthopaedic implants with various surface chemistries and 3-dimensional geometries [7].

However, most orthopaedic implants lack intrinsic osteoinductivity—the capacity to stimulate undifferentiated and pluripotent stem cells to differentiate into the bone-forming cell lineage. Consequently, these materials alone cannot induce bone formation. An effective approach for overcoming this problem is to incorporate osteoinductive drugs. Bone morphogenetic proteins (BMPs) are the cytokines most widely used to confer osteoinductivity [8,9]. Among them, BMP-2, BMP-4, BMP-6, and BMP-7 have long been recognised as osteoinductive and are the most important cytokines in the field of bone tissue engineering. The *in vivo* implantation of exogenous BMPs can induce osteogenesis by blood-borne mesenchymal stem cells (MSCs). In the USA, two products comprising recombinant human (rh) BMP-2 or rhBMP-7 in absorbable collagen have already been approved for clinical application in non-union bone fractures and spinal fusions [10].

In current clinical practice, collagen sponges have been functionalised by the adsorption of several milligrams of BMP-2 (e.g., INFUSE^®^), with the goal of promoting the repair of large bony defects. However, this method of BMP-2 delivery is far from satisfactory because a surface-adsorbed depot of the protein is released too rapidly (in a single high-dose burst) [11,12] to induce a sustained osteogenic response at the site of implantation. This difficulty cannot be overcome simply by increasing the loading dose of BMP-2. Apart from the tremendous expense, the transiently high local concentration of BMP-2 could induce deleterious side effects, such as an over-stimulation of local bone resorption and an induction of bone formation at unintended sites [13,14,15].

To maximise efficacy, BMPs must be delivered to the target site gradually, at a low level and in a sustained manner, rather than in a single high-dose burst [9,16]. Surface coatings have been recognised as an effective way to modify orthopaedic implants and deliver BMPs for the induction of bone formation. Continuous efforts have been devoted to the development of advanced surface coatings to realise the controlled release of BMPs and to maximise their osteoinductive efficacy. In this review, we summarise recent advances in the development of BMP-functionalised coatings to promote osteogenesis for orthopaedic implants.

## 2. BMP (Bone Morphogenetic Protein)

Several growth factors, such as basic fibroblast growth factor (bFGF), insulin-like growth factors (IGFs), transforming growth factor-β (TGF-β), platelet-derived growth factor (PDGF), and vascular endothelial growth factor (VEGF), have been found to promote new bone formation through their effects on the recruitment, proliferation, and differentiation of bone-forming cells and angiogenesis. However, only BMPs can induce new bone formation in a pro-fibrotic microenvironment. BMPs are a group of proteinaceous growth factors in the TGF-β superfamily [17]. The discovery of BMPs in the pioneering work by Urist in 1965 [18] was a landmark in the development of bone tissue engineering. The classical role for BMPs is considered to be the induction of (ectopic) cartilage and bone formation [18,19]. Due to continuous efforts over the past half century, BMPs are currently recognised as a group of metabologens that provides pivotal morphogenetic signals and orchestrates tissue architecture throughout the body [20]. The BMP family consists of more than 30 members [17]. In humans, 19 BMP family members are designated as BMPs. According to their gene homology, protein structure and functions, the 19 members are further subdivided into seven subgroups: BMP-2/4, BMP-3/3b, BMP-5/6/7/8/8b, BMP-9/10, BMP-11/growth and differentiation factor 8 (GDF8), BMP-12/13/14 and BMP-15/GDF9 [10,21]. Most of the mature BMP molecules (except GDF3, GDF9, and GDF9B [22,23]) consist of two monomers that are covalently linked through a disulphide bond [10]. When the two monomers composing one ligand are derived from the same BMP gene, the BMP ligand is termed a “homodimeric BMP” or a BMP homodimer. When the two monomers composing one ligand are derived from different BMP genes, the BMP ligand is termed a “heterodimeric BMP” or a BMP heterodimer. The present knowledge of BMPs is largely based on homodimeric BMPs.

BMPs play pleiotropic roles in promoting the differentiation of pluripotent stem cells along different lineages, e.g., osteogenesis [24], adipogenesis [25] and chondrogenesis [26]. The cellular and therapeutic effects of BMPs are mediated by their downstream signalling pathways, which are initiated by the binding of BMPs to transmembrane serine/threonine kinase receptors. Subsequently, the binding of BMPs triggers specific intracellular signalling pathways that control the transcription of specific target genes [27]. Two types of BMP receptors exist: type I and type II. Type I receptors include activin receptor type-IA (ActR-IA), BMP receptor type-IA (BMPR-IA) and BMP receptor type-IB (BMPR-IB). The type II receptors include BMP receptor type-II (BMPR-II), activin receptor type IIA (ActR-IIA) and activin receptor type IIB (ActR-IIB) [28]. Both types of receptors are indispensable for forming a functional complex to initiate downstream signalling events [29].

**Figure 1:**
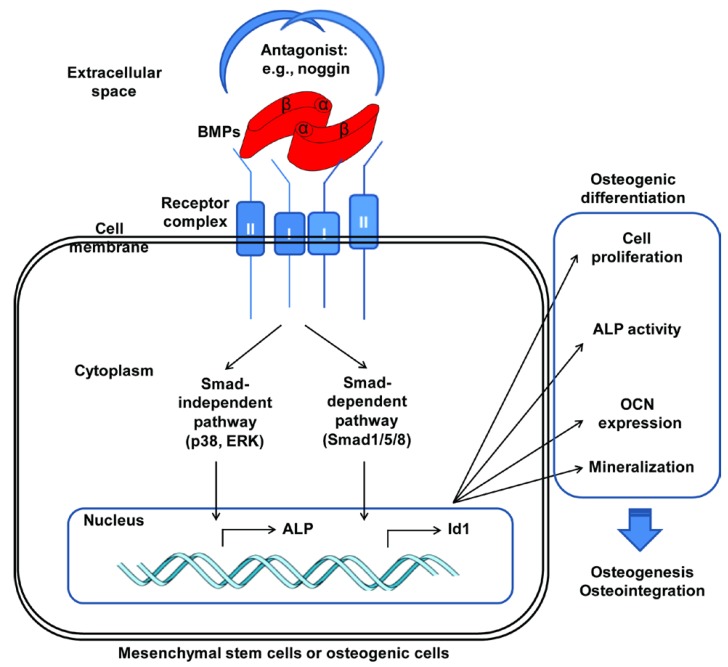
Schematic illustrating the signalling of bone morphogenetic proteins (BMPs) to induce osteogenic differentiation of mesenchymal stem cells or osteogenic cells. ALP: alkaline phosphatase; OCN: osteocalcin; Id: inhibitor of DNA binding 1; ERK: extracellular signal-related kinase.

BMPs can trigger two primary downstream signalling pathways by binding to different receptor complexes: Smad-dependent and Smad-independent signalling pathways [27]. Activated BMP receptors phosphorylate Smad1/5/8, which assembles into a complex with Smad4 and translocates to the nucleus, where it then regulates the transcription of target genes, such as inhibitor of DNA binding 1 (Id1), distal-less homeobox 5 (Dlx 5), runt-related transcription factor 2 (Runx2) and osterix. In addition to Smad-dependent signalling, a series of Smad-independent downstream signalling pathways are also activated, including mitogen-activated protein kinase (MAPK) pathways such as the p38, c-Jun N-terminal kinase (JNK) and extracellular signal-related kinase (ERK) pathways. These pathways play essential roles in BMP-induced osteogenic events [30] (Figure 1). During the process of osteoblastogenesis, mesenchymal stromal cells and osteogenic cells proliferate and express alkaline phosphatase (ALP) and osteocalcin (OCN), ultimately leading to mineralisation and the formation of bone tissue. In addition, highly sulphated and negatively charged glycosaminoglycan (GAG) chains, such as heparin sulphate or chondroitin sulphate, on cell surfaces or in the extracellular matrix can act as co-receptors presenting BMPs to their cell surface signalling receptors [31]. BMP-2-mediated Id1 induction has an intracellular requirement for sulphated molecules [32]. In the presence of heparin, the half-life of BMP-2 in culture medium was prolonged by nearly 20-fold, and a larger amount of bone formation was observed in an *in vivo* model [33]. Mimicking these biological events, a low dose of exogenous 2-*N*, 6-*O*-sulphated chitosan potentiated the osteoinductive activity of BMPs *in vitro* and *in vivo* by promoting BMP signalling pathways [34].

BMP-2, BMP-4, BMP-6, and BMP-7 have long been recognised as osteoinductive, and BMP-2 is the most widely used BMP for conferring osteoinductivity to orthopaedic implants. BMPs have been applied as recombinant proteins or genes. Recently, some BMPs have been reported to possess significantly higher osteoinductive efficiency than BMP-2 or BMP-7. For example, heterodimeric BMP-2/7 has been shown to induce the *in vitro* osteoblastogenesis of pre-osteoblasts more rapidly with a significantly lower concentration threshold and a significantly higher dose-efficiency than homodimeric BMP-2 or BMP-7 [35]. However, a 1:1 mixture of homodimeric BMP-2 and homodimeric BMP-7 did not show a synergistic effect. These phenomena suggest that heterodimeric BMP-2/7 induces bone formation via a specific pattern of signalling pathways [28]. The extracellular antagonist of BMPs—noggin—has shown a reduced antagonism to heterodimeric BMPs compared to homodimeric BMPs [36]. Moreover, heterodimeric BMPs have been shown to more rapidly promote the formation of bone with a more mature microarchitecture *in vivo* in a peri-implant bone defect compared to homodimeric BMPs [37]. BMP-9 was also recently shown to be superior to BMP-2 and BMP-7 in inducing the osteogenesis of MSCs. BMP-9 does not have a dimerised structure. Its signalling pathways are primarily mediated by receptors comprising ActR-IA and activin receptor-like kinase 1 [38]. The latter is known to be a receptor for TGF-β. In addition, BMP-9 has also been shown to resist the inhibitory effect of noggin and potently induce the osteogenic differentiation of mesenchymal cells [39]. These findings indicate that heterodimeric BMPs and BMP-9 are promising for inducing osteogenesis for orthopaedic implants.

However, when used in the clinically available forms and doses, BMPs have been shown to cause a series of adverse side effects, such as pain, radiculitis, ectopic bone formation, osteolysis, and poor global outcomes [40]. In addition, higher doses of rhBMP-2 have been associated with a greater apparent risk of new malignancy. Most of these adverse effects may be associated with supraphysiological doses (several milligrams) of BMPs. In recent years, we have been pleased to see advanced materials that enable the controlled release and, thus, the osteoinductive function of BMPs in physiological doses (several micrograms). BMPs at physiological doses are expected to cause significantly fewer adverse effects in comparison to supraphysiological doses. Consequently, advanced materials, including various coatings, have been developed to exert the osteoinductive functions of BMPs at physiological doses, thus conferring BMPs a promising application potential.

## 3. Adsorption of BMPs

**Figure 2:**
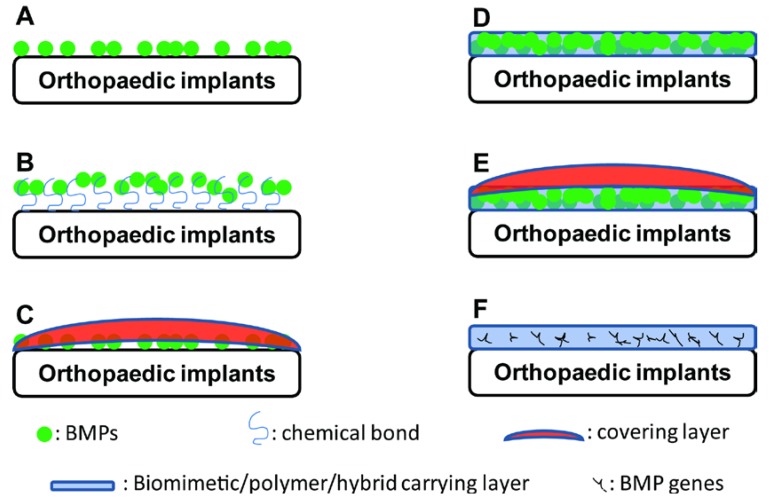
Schematic illustrating the types of BMP/BMP-functionalised coatings on orthopaedic implants. (**A**) Direct adsorption; (**B**) Immobilisation of BMP through a chemical bond; (**C**) Adsorption of BMPs with a covering/barrier layer; (**D**) Incorporation of BMPs into a biomimetic/polymer/hybrid carrying layer; (**E**) Incorporation of BMPs into a polymer/hybrid carrier layer in combination with a covering/barrier layer; and (**F**) Incorporation of BMP genes into a polymer/hybrid carrier layer.

The classical method for applying recombinant BMPs is to form a coating layer of BMPs by superficially adsorbing them onto collagen sponges without additional bonding (Figure 2A). With this method, a large proportion of BMPs is released in a short time after exposure to the physiological milieu. The released BMPs can be rapidly deactivated by enzymes in the body. Consequently, a supraphysiological amount (e.g., milligrams) of BMPs must be applied to elicit osteoinductive effects [41]. Huh *et al.* [42] used 0.75 and 1.5 mg/mL of BMP-2 to functionalise dental implants, and a significantly higher volume of vertically formed bone and improved implant stability were achieved. However, this delivery method also raises concerns of possible side effects, such as over-stimulation of local bone resorption [15].

Attempts have been made at immobilising BMPs to both enhance the adsorption efficiency and enable slow release. Immobilisation of BMPs on orthopaedic implants is critical for improving stem cell-mediated bone tissue engineering. Shiels *et al.* [43] demonstrated that BMP-2 can be bonded to the surface of hydroxyapatite through polyethyleneimine (PEI) and a polyethylene glycol (PEG) tether. Although slow release of BMP-2 was achieved by this method, the amount of BMP-2 loaded (37.0 ± 7.7 ng/cm^2^) was insufficient to facilitate more substantial bone regeneration *in vivo* [44]. In contrast, oxygen termination of nanocrystalline diamond can strongly immobilise BMP-2 and can enhance the osteointegration of dental implants [45].

The benefits of coatings may arise not only from enhancing the incorporation rate or slowing the release of BMP-2 but also from maintaining the bioactivity of BMP-2. La *et al.* [46] coated titanium substrates with grapheme oxide (GO) through a layer-by-layer (LbL) assembly of positively (GO-NH_3_^+^) and negatively (GO-COO^−^) charged GO sheets. BMP-2 was loaded on the GO-coated Ti substrate with the outermost coating layer of GO-COO^−^ (Ti/GO^−^). In comparison with native BMP-2, significant conformational changes were found in the BMP-2 that was directly adsorbed on Ti but not on Ti/GO^−^. Furthermore, BMP-2 adsorbed onto Ti/GO^−^ induced more robust phosphorylation of Smad 1/5/8 than when it was either adsorbed onto bare Ti or assayed in solution. The higher bioactivity of BMP-2 may be attributed to the conformational stability conferred by immobilisation on Ti/GO^−^ [46].

In comparison to multistep strategies, a biologically inspired one-step strategy based on polymerised dopamine was introduced to immobilise BMPs or BMP-derived peptides on both polymeric [47] and titanium implants [48] (Figure 2B). The predeposition of a polymerised dopamine layer facilitates highly efficient, facile immobilisation of BMPs and BMP-derived peptides. Orthopaedic implants functionalised by this method were shown to greatly enhance the *in vitro* osteogenic differentiation and calcium mineralisation of both human bone marrow-derived MSCs (BMSCs) and adipose tissue-derived stem cells (ASCs) [47,48]. Furthermore, transplanted ASCs on these functionalised scaffolds significantly promoted *in vivo* bone formation in critical-sized calvarial bone defects [47].

Furthermore, the immediate exposure of adsorbed BMP-2 to the physiological milieu can be avoided by using a covering layer (Figure 2C). Kim *et al.* [49] used a cross-linked alginate covering layer to enable the slow release of BMP-2 or an osteoinductive peptide from a polycaprolactone scaffold. Peterson *et al.* [50] prepared a polyelectrolyte coating of poly-l-histidine and poly(methacrylic acid), which was shown to be effective for the sustained release of negatively charged species under physiological conditions. This complex demonstrated pH-dependent release, with a maximum release at pH = 5–6 and low levels of sustained release at pH = 7–8. A reduced initial burst release and higher amounts of sustained release were observed when lower-molecular-weight poly(methacrylic acid) was used.

Moreover, when a calcium phosphate carrier is used, a prolonged retention of growth factors is always observed, which may result in reduced osteoinductive activity [51,52]. Column chromatography separation techniques have demonstrated that Ca-P ceramics exhibit a high-binding affinity for proteins [53]. Thus, for these carriers, a strategy is needed to accelerate the release of BMPs. Zhao *et al.* [54] used negatively charged chitosan, sulphated chitosan, to accelerate the release of BMP-2 from a calcium-deficient hydroxyapatite scaffold. An almost twofold increase in the release percentage was achieved using the sulphated chitosan coating. Ionic interactions between the positively charged BMP-2 and the negatively charged polysaccharide chains increase the affinity of sulphated chitosan for BMP-2, which could significantly enhance BMP-2 release from Ca-P ceramics. As a result, BMP-2 with a sulphated chitosan coating resulted in more extensive new bone formation in comparison with controls.

## 4. BMP-Carrying Coatings

A liquid-solid transition can enable bioactive agents to be encapsulated with a 100% incorporation rate, allowing organic molecules to be employed as a very simple and efficient coating strategy for different orthopaedic implants (Figure 2D). With the degradation of the coating materials, the bioactive agents can be slowly released into the surrounding microenvironment.

Synthetic polymeric materials can be dissolved in organic solvents, in which bioactive agents can be incorporated. A liquid-solid transition of polymeric materials can be realised through the evaporation of the organic solvents. Poly(d,l-lactide) (PDLLA) is one of the most widely used polymeric materials for bone tissue engineering. PDLLA has been dissolved in a volatile solvent (chloroform) to enable the incorporation of growth factors (IGF-I and TGF-β1) and coating formation on metal wires [55]. A slow release of the growth factors was achieved. The same strategy was later applied to functionalise titanium Kirschner wires by incorporating BMP-2 [56]. The BMP-2-PDLLA coating-functionalised titanium Kirschner wires were shown to rescue delayed osteotomy healing in a clinically relevant animal model [56]. By changing the concentration and coating sequence of PDLLA, a sequential release of two (gentamicin and BMP-2) or three components (gentamicin, insulin-like growth factor I and BMP-2) was achieved to sequentially exert antibacterial, osteopromotive and osteoinductive functions [56]. The activity of the early release of gentamicin from the two-layer coating was confirmed microbiologically. The subsequently released BMP-2 stimulated the metabolic activity and alkaline phosphatase (ALP) activity of C2C12 cells after 2 weeks. In the three-layer-coated wires, insulin-like growth factor I continuously stimulated cell proliferation, while BMP-2 enhanced ALP activity between 1–3 weeks. The sequential release of growth factors revealed an additive effect on the metabolic activity and ALP expression of primary osteoblast-like cells compared to single coated controls.

The use of organic solvents may present a potential risk of harming the host tissues and triggering an undesired foreign-body-giant-cell response. Furthermore, organic solvents may compromise the bioactivity of proteinaceous bioactive agents. In comparison, water-soluble organic molecules are advantageous for preserving the bioactivity of proteins such as BMPs. Fibrin is a natural water-soluble organic molecule that can be polymerised by thrombin. The process of fibrin polymerisation is similar to the clotting of blood, in which the bioactivity of proteinaceous cytokines is retained. The liquid-solid transition of polymerised fibrin can be achieved simply by drying under vacuum. Kang *et al.* [57] functionalised a solid freeform-based scaffold with a BMP-2-embedded fibrin/hyaluronic acid coating. The fibrin/hyaluronic acid coating significantly enhanced initial cell attachment. Furthermore, the *in vitro* release of BMP-2 from the fibrin/hyaluronic acid-coated scaffolds was sustained for 3 days. The sustained release stimulated the ALP activity of ASCs seeded on the scaffold for 10 days to a greater degree in comparison to soluble BMP-2 that was added to the culture media. Importantly, the transplantation of undifferentiated ASCs seeded on BMP-2-loaded, fibrin/hyaluronic acid-coated scaffolds resulted in improved bone formation and mineralisation compared to undifferentiated ASCs seeded on uncoated scaffolds as well as on fibrin/HA-coated scaffolds without BMP-2 (but containing BMP-2 in the cell suspension medium). The same principle can also be applied to produce a BMP-functionalised coating with other organic molecules, such as gelatin [58] and chitosan [59].

Layer-by-Layer (LbL) fabrication, in which a charged substrate is alternately dipped in positively and negatively charged polymer baths to build a nanolayered thin film, is another important technique for creating coating layers with embedded BMP-2. Polyelectrolyte multilayer films are highly attractive as ultrathin biological reservoirs because of the ability to conformally coat difficult geometries, the use of aqueous processing, which is likely to preserve the function of fragile proteins, and the tunability of incorporation and release profiles [60,61]. Macdonald *et al.* [62] created an ultrathin polyelectrolyte multilayer film by repeatedly dipping a scaffold in four solutions containing (1) poly(β-aminoester) (positively charged); (2) chondroitin sulphate (negatively charged); (3) BMP-2 (positively charged); and (4) chondroitin sulphate. This tetralayer structure was repeated 100 times for all LbL films in this communication. BMP-2 released from the polyelectrolyte multilayer films retained its ability to induce both *in vitro* osteogenic differentiation and *in vivo* bone formation intramuscularly. Guillot *et al.* [63] developed an osteoinductive coating on a porous titanium implant using biomimetic polyelectrolyte multilayer films loaded with BMP-2. The amount of BMP-2 loaded in these films was tuned over a large range by varying the extent of cross-linking of the film and the initial concentration of BMP-2. An important property of this coating is that it was shown to preserve the bioactivity of BMP-2 in resistance to various adverse conditions, such as long-term storage and γ-irradiation sterilisation, as shown by *in vitro* data. Van den Beucken investigated three loading modalities of BMP-2 [superficial (s), deep (d), and double-layer (dl)] in multilayer coatings created using cationic poly-d-lysine or poly(allylamine hydrochloride) and anionic deoxyribonucleic acid (DNA) [64]. All of the differently loaded multilayered DNA-based coatings showed an initial burst release followed by an incremental sustained release of the remaining BMP-2. In *in vitro* experiments, the superficially loaded and double-layer-loaded coatings significantly accelerated calcium deposition. In contrast, the d-loaded multilayered DNA-based coatings influenced osteoblast-like cell behaviour by decreasing the deposition of calcium. Consequently, the loading modality may also significantly influence the efficacy of BMP-2.

Using the LbL technique, Min *et al.* [58] presented a new strategy—the implementation of laponite clay barriers (Figure 2E). The barrier layer brings two benefits: (1) it allows for physical separation of the two components (gentamicin and BMP-2) by controlling interlayer diffusion; (2) it leads to a significant reduction in the release dose and an increase in the release timescale. This new platform for multi-drug localised delivery can be easily fabricated, tuned, and translated to a variety of implant applications in which control over the spatial and temporal release profiles of multiple drugs is desired.

## 5. BMP-Functionalised Biomimetic Coatings

In 1990, Kokubo and colleagues, for the first time, introduced the concept of biomimetic mineralisation [65]. By this method, materials can be coated with a layer of apatite by immersion into simulated body fluid (SBF)—a solution with ion concentrations that are approximately equal to those of human blood plasma. The layer of calcium phosphate that is produced can promote the differentiation of bone-marrow stromal cells into osteoblasts [66], enhance bone ingrowth and bone-implant contact [67,68,69], and reduce fibrous encapsulation [70]. However, the application of the original biomimetic technique was limited due to its two intrinsic disadvantages: a long immersion period (approximately 1–2 weeks) and the need for active chemical groups for the formation of the apatite layer. Active chemical groups, such as dihydrogen phosphate (H_2_PO_4_) or carboxylic acid (COOH) moieties, are highly conducive to biomimetic mineralisation, while those composed of methyl groups (CH_3_) are unpropitious for this process [71]. Because many of the commercially available orthopaedic implants lack such active chemical groups on their surfaces, additional treatments must be performed to enable the coating formation [72].

To overcome this limitation, a two-phase biomimetic coating technique was developed. This two-phase biomimetic coating process involves the formation of an initial amorphous layer as a seeding layer, followed by the subsequent deposition of a crystalline protein-carrying layer. Without the need for additional surface modifications, this technique has been used to biomimetically coat various orthopaedic implants with different physicochemical properties, including titanium [73,74,75], deproteinised bovine bone [76], collagen and three synthetic polymeric materials [77], within 3 days. The morphological and physicochemical properties of the coatings have been shown to be independent of the surface chemistry, the surface geometry and the three-dimensional structure of the underlying materials [77]. The broad applicability of the two-phase biomimetic coating can be attributed to the amorphous seeding layer because tiny particles of amorphous calcium phosphate, which are formed under the nucleation-inhibitory influence of Mg^2+^ [78] and HCO3^2−^ [79], can be captured and immobilised on the substratum through mechanical gomphosis [77]. These amorphous calcium phosphate spheres then serve as nucleation sites for the subsequent growth of a crystalline lattice of calcium phosphate [80]. With this two-phase biomimetic coating, BMPs can be added to the coating solutions and co-precipitated with the crystalline calcium phosphate to form a BMP-functionalised biomimetic coating. The crystalline latticework of the biomimetic coating provides a three-dimensional reservoir to store and release BMPs gradually [81] and in a cell-mediated manner over a period of several weeks [82]. Significantly higher volume densities of newly formed bone tissue have been consistently induced by biomimetic coatings incorporating BMP-2 for metallic implants [81,83], deproteinised bovine bone [76] and four types of polymers [84] compared to materials with similar amounts of adsorbed BMP-2.

Interestingly, the bone formation induced by coatings incorporating BMP-2 shows a unique characteristic: the volume density of the newly formed bone is proportional to the initial surface-area density of the orthopaedic implants [84]. This result indicates that polymers with a more dense surface area will be associated with a higher volume density of bone. This ossification may provide an explanation for the dependence of bone formation on the surface-area density of the functionalised materials. Thus, this technique challenges current approaches in tissue engineering in which pore size and porosity are heavily emphasised [85]. After a 5-week implantation, significantly lower volume densities of foreign-body giant cells (FBGCs) were associated with coatings incorporating BMP-2 compared to orthopaedic implants either alone, with adsorbed BMP-2 or with a coating only. These findings indicated that the biomimetic coating incorporating BMP-2 not only induced and sustained bone formation with a higher efficiency but also reduced the host inflammatory response, such as the formation of FBGCs.

One limitation of biomimetic coatings is that the protein incorporation rate is relatively low (3%–15%) [74,86,87]. This low incorporation may lead to a waste of expensive BMPs and may limit their clinical applications. Yu *et al.* [88] attempted to improve the protein incorporation rate by carefully adjusting the substrate surface area to SBF volume ratio (SSA/SV ratio). The authors achieved a very high incorporation rate (90%) of bovine serum albumin when the ratio of the substrate surface area to SBF volume was as high as 0.072.

## 6. Coatings for the Delivery of BMP Genes

Although growth factor-based bone regeneration has been widely used in clinical practice, the biological activity of the soluble factors that promote bone formation *in vivo* can be limited by diffusion and degradation. To address these problems, new approaches based on the delivery of genes that encode these growth factors to the target cells have been established. In these approaches, the transfected cells serve as local “bioreactors”, as they express the exogenous genes and secrete the synthesised proteins into their local microenvironment [89].

Naked plasmid DNA physically entrapped in a polymer matrix sponge has been associated with low transfection efficiencies [90]. In the past decade, several advanced vectors have been developed to deliver BMP genes with high transfection efficiency. An ideal vector would possess the following characteristics: avoidance of an immunological host response, preferential binding to specific target cells, transduction of dividing and non-dividing cells, no disruption of normal cell function, expression of genes at an appropriate therapeutic level, ability to allow for external control of protein expression, and ease of production at a reasonable cost [91,92,93]. Vector systems can be classified into non-viral and viral vectors. Both types of vectors have their respective advantages and disadvantages. Viral vectors, such as retroviruses, lentiviruses, adenoviruses and adeno-associated viruses (AAV), show relatively higher transfection efficiency [94], but may cause immunological rejection and can disrupt normal gene functions [95,96]. Non-viral vectors, such as DNA plasmids, lipoplexes, and polyplexes, can avoid many of the problems associated with viral vectors, but they are associated with DNA instability, inefficient delivery to target cells, variable clearance by lysosomes, unpredictable cytosolic transport, and inconsistent transcription of the desired genes [97]. The choice of a vector for gene therapy depends on the desired duration of protein function, anatomical location, condition to be treated, and the desire for an *in vivo* or *ex vivo* approach [91].

### 6.1. Coatings with Non-Viral Vectors Delivering BMP Genes

Kolk *et al.* [98] published a strategy for establishing a gene-activated matrix on titanium using gene vectors protected by a polylactide coating (Figure 2F). Copolymer-protected gene vectors were prepared by lyophilising a mixture of polyethylenimine (PEI), plasmid DNA and the negatively charged protective copolymer P6YE5C. The polyplex was then lyophilised into dried DNA complexes. The complexes were suspended in PDLLA and coated onto titanium surfaces. The vector release, cell viability and gene transfer efficiency to NIH 3T3 fibroblasts were strongly dependent on the vector dose and its ratio to the PDLLA film thickness.

Jiang *et al.* [99] fabricated a multilayered cationic coating of hyaluronic acid/liposome-DNA complexes (HA/LDc) on titanium using a LbL assembly approach and evaluated it as a delivery vehicle for recombinant human BMP-2. Cells that were seeded on the HA/LDc coating secreted a significantly higher amount of BMP-2 into the culture medium than pre-osteoblasts (MC3T3-E1 cell line) seeded directly onto the titanium surface or onto a coating without BMP plasmid at 3 days. This coating also led to significantly higher ALP activity in MC3T3-E1 cells than the controls after 7 and 14 days of culture. Thus, it was concluded that pre-osteoblasts cultured on the multilayer HA/LDc coating surface could secret rhBMP-2 protein at levels that were effective in inducing early osteogenic differentiation.

### 6.2. Coatings with Viral Vectors Delivering BMP Genes

Chen *et al.* [100] prepared a type I collagen-avidin coating on titanium. Adenoviral vectors expressing BMP-7 (Ad-BMP-7) were attached to the coating through hexon-specific antibodies. The anti-adenohexon antibody adhered strongly to the collagen-avidin gels. BMP-7 gene expression was precisely localised to cells growing on the gels functionalised with the hexon-specific antibody. Osteoblasts on the titanium delivering Ad-BMP-7 exhibited higher ALP activity than the control condition without Ad-BMP-7.

AAVs are also one of the most widely used viral vectors. A conventional AAV vector has a rate-limiting step that involves second-strand synthesis, as the typical AAV genome is a single-stranded DNA template. To promote the transfection efficiency of AAV vectors, a self-complementary AAV (scAAV) was developed [101]. Rather than waiting for cell-mediated synthesis of the second strand, upon infection, the two complementary halves of the scAAV will associate to form one double-stranded DNA (dsDNA) unit that is ready for immediate replication and transcription. Collagen coatings carrying scAAV-BMP-2 have been found to significantly enhance BMP-2 production and alkaline phosphatase activity of human MSCs in two-dimensional cultures [102]. Furthermore, acellular scAAV-BMP-2-coated three-dimensional porous poly(ε-caprolactone) scaffolds have been shown to increase bony bridging and induce significantly higher bone ingrowth and mechanical properties compared to controls in critical-sized femoral defects in immunocompromised rats. Similar results were also achieved using scAAV-BMP-2-coated allografts [103].

## 7. Conclusions

BMPs, particularly BMP-2, are highly osteoinductive and induce *in vitro* osteoblastogenesis and *in vivo* osteogenesis. Osteoinductivity can be conferred to orthopaedic implants with six types of BMP-functionalised coatings: (A) direct adsorption; (B) immobilisation of BMP through chemical bonding; (C) adsorption of BMPs with a covering layer; (D) incorporation of BMPs into a polymer/hybrid carrier/barrier layer; (E) incorporation of BMPs into a polymer/hybrid carrier layer in combination with a covering/barrier layer; and (F) incorporation of BMP genes into a polymer/hybrid carrier layer (Figure 2). The latter five types of BMP-functionalised coatings can result in slow, localised release of BMPs, thereby significantly enhancing osteogenesis and the osteointegration of orthopaedic implants using a low dose of BMPs.

## References

[1] Claffey N., Clarke E., Polyzois I., Renvert S. (2008). Surgical treatment of peri-implantitis. J. Clin. Periodontol..

[2] Saito N., Takaoka K. (2003). New synthetic biodegradable polymers as BMP carriers for bone tissue engineering. Biomaterials.

[3] Kretlow J.D., Mikos A.G. (2007). Review: Mineralization of synthetic polymer scaffolds for bone tissue engineering. Tissue Eng..

[4] Silber J.S., Anderson D.G., Daffner S.D., Brislin B.T., Leland J.M., Hilibrand A.S., Vaccaro A.R., Albert T.J. (2003). Donor site morbidity after anterior iliac crest bone harvest for single-level anterior cervical discectomy and fusion. Spine.

[5] Heary R.F., Schlenk R.P., Sacchieri T.A., Barone D., Brotea C. (2002). Persistent iliac crest donor site pain: Independent outcome assessment. Neurosurgery.

[6] Orthopedic Implants. http://orthopedicimplants.wordpress.com/.

[7] Mantripragada V.P., Lecka-Czernik B., Ebraheim N.A., Jayasuriya A.C. (2013). An overview of recent advances in designing orthopedic and craniofacial implants. J. Biomed. Mater. Res. A.

[8] Carson J.S., Bostrom M.P. (2007). Synthetic bone scaffolds and fracture repair. Injury.

[9] Sokolsky-Papkov M., Agashi K., Olaye A., Shakesheff K., Domb A.J. (2007). Polymer carriers for drug delivery in tissue engineering. Adv. Drug Deliv. Rev..

[10] Bessa P.C., Casal M., Reis R.L. (2008). Bone morphogenetic proteins in tissue engineering: The road from the laboratory to the clinic, part I (basic concepts). J. Tissue Eng. Regen. Med..

[11] Haidar Z.S., Hamdy R.C., Tabrizian M. (2009). Delivery of recombinant bone morphogenetic proteins for bone regeneration and repair. Part A: Current challenges in BMP delivery. Biotechnol. Lett..

[12] Haidar Z.S., Hamdy R.C., Tabrizian M. (2009). Delivery of recombinant bone morphogenetic proteins for bone regeneration and repair. Part B: Delivery systems for BMPs in orthopaedic and craniofacial tissue engineering. Biotechnol. Lett..

[13] Shields L.B., Raque G.H., Glassman S.D., Campbell M., Vitaz T., Harpring J., Shields C.B. (2006). Adverse effects associated with high-dose recombinant human bone morphogenetic protein-2 use in anterior cervical spine fusion. Spine.

[14] Smith D.M., Cooper G.M., Mooney M.P., Marra K.G., Losee J.E. (2008). Bone morphogenetic protein 2 therapy for craniofacial surgery. J. Craniofac. Surg..

[15] Toth J.M., Boden S.D., Burkus J.K., Badura J.M., Peckham S.M., McKay W.F. (2009). Short-term osteoclastic activity induced by locally high concentrations of recombinant human bone morphogenetic protein-2 in a cancellous bone environment. Spine.

[16] Lutolf M.P., Weber F.E., Schmoekel H.G., Schense J.C., Kohler T., Muller R., Hubbell J.A. (2003). Repair of bone defects using synthetic mimetics of collagenous extracellular matrices. Nat. Biotechnol..

[17] Ducy P., Karsenty G. (2000). The family of bone morphogenetic proteins. Kidney Int..

[18] Urist M.R. (1965). Bone: Formation by autoinduction. Science.

[19] Wang E.A., Rosen V., Cordes P., Hewick R.M., Kriz M.J., Luxenberg D.P., Sibley B.S., Wozney J.M. (1988). Purification and characterization of other distinct bone-inducing factors. Proc. Natl. Acad. Sci. USA.

[20] Reddi A.H., Reddi A. (2009). Bone morphogenetic proteins (BMPs): From morphogens to metabologens. Cytokine Growth Factor Rev..

[21] Reddi A.H. (2005). BMPs: From bone morphogenetic proteins to body morphogenetic proteins. Cytokine Growth Factor Rev..

[22] Liao W.X., Moore R.K., Otsuka F., Shimasaki S. (2003). Effect of intracellular interactions on the processing and secretion of bone morphogenetic protein-15 (BMP-15) and growth and differentiation factor-9. Implication of the aberrant ovarian phenotype of BMP-15 mutant sheep. J. Biol. Chem..

[23] Sieber C., Kopf J., Hiepen C., Knaus P. (2009). Recent advances in BMP receptor signaling. Cytokine Growth Factor Rev..

[24] Levi B., Hyun J.S., Nelson E.R., Li S., Montoro D.T., Wan D.C., Jia F.J., Glotzbach J.C., James A.W., Lee M. (2011). Nonintegrating knockdown and customized scaffold design enhances human adipose-derived stem cells in skeletal repair. Stem Cells.

[25] Tseng Y.H., Kokkotou E., Schulz T.J., Huang T.L., Winnay J.N., Taniguchi C.M., Tran T.T., Suzuki R., Espinoza D.O., Yamamoto Y. (2008). New role of bone morphogenetic protein 7 in brown adipogenesis and energy expenditure. Nature.

[26] Kim H.J., Im G.I. (2009). Combination of transforming growth factor-beta2 and bone morphogenetic protein 7 enhances chondrogenesis from adipose tissue-derived mesenchymal stem cells. Tissue Eng. A.

[27] Derynck R., Zhang Y.E. (2003). Smad-dependent and Smad-independent pathways in TGF-beta family signalling. Nature.

[28] Guo J., Wu G. (2012). The signaling and functions of heterodimeric bone morphogenetic proteins. Cytokine Growth Factor Rev..

[29] Miyazono K., Kamiya Y., Morikawa M. (2010). Bone morphogenetic protein receptors and signal transduction. J. Biochem..

[30] Xiao G., Gopalakrishnan R., Jiang D., Reith E., Benson M.D., Franceschi R.T. (2002). Bone morphogenetic proteins, extracellular matrix, and mitogen-activated protein kinase signaling pathways are required for osteoblast-specific gene expression and differentiation in MC3T3-E1 cells. J. Bone Miner. Res..

[31] Takada T., Katagiri T., Ifuku M., Morimura N., Kobayashi M., Hasegawa K., Ogamo A., Kamijo R. (2003). Sulfated polysaccharides enhance the biological activities of bone morphogenetic proteins. J. Biol. Chem..

[32] Osses N., Gutierrez J., Lopez-Rovira T., Ventura F., Brandan E. (2006). Sulfation is required for bone morphogenetic protein 2-dependent Id1 induction. Biochem. Biophs. Res. Commun..

[33] Zhao B., Katagiri T., Toyoda H., Takada T., Yanai T., Fukuda T., Chung U.I., Koike T., Takaoka K., Kamijo R. (2006). Heparin potentiates the *in vivo* ectopic bone formation induced by bone morphogenetic protein-2. J. Biol. Chem..

[34] Zhou H., Qian J., Wang J., Yao W., Liu C., Chen J., Cao X. (2009). Enhanced bioactivity of bone morphogenetic protein-2 with low dose of 2-*N*, 6-*O*-sulfated chitosan *in vitro* and *in vivo*. Biomaterials.

[35] Zheng Y., Wu G., Zhao J., Wang L., Sun P., Gu Z. (2010). rhBMP2/7 heterodimer: An osteoblastogenesis inducer of not higher potency but lower effective concentration compared with rhBMP2 and rhBMP7 homodimers. Tissue Eng. A.

[36] Zhu W., Kim J., Cheng C., Rawlins B.A., Boachie-Adjei O., Crystal R.G., Hidaka C. (2006). Noggin regulation of bone morphogenetic protein (BMP) 2/7 heterodimer activity *in vitro*. Bone.

[37] Wang J., Zheng Y., Zhao J., Liu T., Gao L., Gu Z., Wu G. (2012). Low-dose rhBMP2/7 heterodimer to reconstruct peri-implant bone defects: A micro-CT evaluation. J. Clin. Periodontol..

[38] Luo J., Tang M., Huang J., He B.C., Gao J.L., Chen L., Zuo G.W., Zhang W., Luo Q., Shi Q. (2010). TGFbeta/BMP type I receptors ALK1 and ALK2 are essential for BMP9-induced osteogenic signaling in mesenchymal stem cells. J. Biol. Chem..

[39] Wang Y., Hong S., Li M., Zhang J., Bi Y., He Y., Liu X., Nan G., Su Y., Zhu G. (2013). Noggin resistance contributes to the potent osteogenic capability of BMP9 in mesenchymal stem cells. J. Orthop. Res..

[40] Carragee E.J., Hurwitz E.L., Weiner B.K. (2011). A critical review of recombinant human bone morphogenetic protein-2 trials in spinal surgery: Emerging safety concerns and lessons learned. Spine J..

[41] Park D.K., Kim S.S., Thakur N., Boden S.D. (2013). Use of recombinant human bone morphogenetic protein-2 with local bone graft instead of iliac crest bone graft in posterolateral lumbar spine arthrodesis. Spine.

[42] Huh J.B., Park C.K., Kim S.E., Shim K.M., Choi K.H., Kim S.J., Shim J.S., Shin S.W. (2011). Alveolar ridge augmentation using anodized implants coated with Escherichia coli-derived recombinant human bone morphogenetic protein 2. Oral Surg. Oral Med. Oral Pathol. Oral Radiol. Endod..

[43] Shiels S.M., Solomon K.D., Pilia M., Appleford M.R., Ong J.L. (2012). BMP-2 tethered hydroxyapatite for bone tissue regeneration: Coating chemistry and osteoblast attachment. J. Biomed. Mater. Res. A.

[44] Shiels S., Oh S., Bae C., Guda T., Singleton B., Dean D.D., Wenke J.C., Appleford M.R., Ong J.L. (2012). Evaluation of BMP-2 tethered polyelectrolyte coatings on hydroxyapatite scaffolds *in vivo*. J. Biomed. Mater. Res. B.

[45] Kloss F.R., Gassner R., Preiner J., Ebner A., Larsson K., Hachl O., Tuli T., Rasse M., Moser D., Laimer K. (2008). The role of oxygen termination of nanocrystalline diamond on immobilisation of BMP-2 and subsequent bone formation. Biomaterials.

[46] La W.G., Park S., Yoon H.H., Jeong G.J., Lee T.J., Bhang S.H., Han J.Y., Char K., Kim B.S. (2013). Delivery of a therapeutic protein for bone regeneration from a substrate coated with graphene oxide. Small.

[47] Ko E., Yang K., Shin J., Cho S.W. (2013). Polydopamine-assisted osteoinductive peptide immobilization of polymer scaffolds for enhanced bone regeneration by human adipose-derived stem cells. Biomacromolecules.

[48] Chien C.Y., Tsai W.B. (2013). Poly(dopamine)-assisted immobilization of Arg-Gly-Asp peptides, hydroxyapatite, and bone morphogenic protein-2 on titanium to improve the osteogenesis of bone marrow stem cells. ACS Appl. Mater. Interfaces.

[49] Kim M., Jung W.K., Kim G. (2013). Bio-composites composed of a solid free-form fabricated polycaprolactone and alginate-releasing bone morphogenic protein and bone formation peptide for bone tissue regeneration. Bioprocess Biosyst. Eng..

[50] Peterson A.M., Mohwald H., Shchukin D.G. (2012). pH-controlled release of proteins from polyelectrolyte-modified anodized titanium surfaces for implant applications. Biomacromolecules.

[51] Ruhe P.Q., Boerman O.C., Russel F.G., Spauwen P.H., Mikos A.G., Jansen J.A. (2005). Controlled release of rhBMP-2 loaded poly(dl-lactic-*co*-glycolic acid)/calcium phosphate cement composites *in vivo*. J. Control. Release.

[52] Ginebra M.P., Traykova T., Planell J.A. (2006). Calcium phosphate cements as bone drug delivery systems: A review. J. Control. Release.

[53] Ruhe P.Q., Kroese-Deutman H.C., Wolke J.G., Spauwen P.H., Jansen J.A. (2004). Bone inductive properties of rhBMP-2 loaded porous calcium phosphate cement implants in cranial defects in rabbits. Biomaterials.

[54] Zhao J., Shen G., Liu C., Wang S., Zhang W., Zhang X., Ye D., Wei J., Zhang Z., Jiang X. (2012). Enhanced healing of rat calvarial defects with sulfated chitosan-coated calcium-deficient hydroxyapatite/bone morphogenetic protein 2 scaffolds. Tissue Eng. A.

[55] Schmidmaier G., Wildemann B., Stemberger A., Haas N.P., Raschke M. (2001). Biodegradable poly(D,L-lactide) coating of implants for continuous release of growth factors. J. Biomed. Mater. Res..

[56] Strobel C., Bormann N., Kadow-Romacker A., Schmidmaier G., Wildemann B. (2011). Sequential release kinetics of two (gentamicin and BMP-2) or three (gentamicin, IGF-I and BMP-2) substances from a one-component polymeric coating on implants. J. Control. Release.

[57] Kang S.W., Kim J.S., Park K.S., Cha B.H., Shim J.H., Kim J.Y., Cho D.W., Rhie J.W., Lee S.H. (2011). Surface modification with fibrin/hyaluronic acid hydrogel on solid-free form-based scaffolds followed by BMP-2 loading to enhance bone regeneration. Bone.

[58] Zhang Q., Tan K., Zhang Y., Ye Z., Tan W.S., Lang M. (2014). *In situ* controlled release of rhBMP-2 in gelatin-coated 3D porous poly(epsilon-caprolactone) scaffolds for homogeneous bone tissue formation. Biomacromolecules.

[59] Jun S.H., Lee E.J., Jang T.S., Kim H.E., Jang J.H., Koh Y.H. (2013). Bone morphogenic protein-2 (BMP-2) loaded hybrid coating on porous hydroxyapatite scaffolds for bone tissue engineering. J. Mater. Sci. Mater. Med..

[60] Macdonald M.L., Rodriguez N.M., Shah N.J., Hammond P.T. (2010). Characterization of tunable FGF-2 releasing polyelectrolyte multilayers. Biomacromolecules.

[61] Wood K.C., Chuang H.F., Batten R.D., Lynn D.M., Hammond P.T. (2006). Controlling interlayer diffusion to achieve sustained, multiagent delivery from layer-by-layer thin films. Proc. Natl. Acad. Sci. USA.

[62] Macdonald M.L., Samuel R.E., Shah N.J., Padera R.F., Beben Y.M., Hammond P.T. (2011). Tissue integration of growth factor-eluting layer-by-layer polyelectrolyte multilayer coated implants. Biomaterials.

[63] Guillot R., Gilde F., Becquart P., Sailhan F., Lapeyrere A., Logeart-Avramoglou D., Picart C. (2013). The stability of BMP loaded polyelectrolyte multilayer coatings on titanium. Biomaterials.

[64] Van den Beucken J.J., Walboomers X.F., Boerman O.C., Vos M.R., Sommerdijk N.A., Hayakawa T., Fukushima T., Okahata Y., Nolte R.J., Jansen J.A. (2006). Functionalization of multilayered DNA-coatings with bone morphogenetic protein 2. J. Control. Release.

[65] Kokubo T., Ito S., Huang Z.T., Hayashi T., Sakka S., Kitsugi T., Yamamuro T. (1990). Ca, P-rich layer formed on high-strength bioactive glass-ceramic A-W. J. Biomed. Mater. Res..

[66] Ohgushi H., Caplan A.I. (1999). Stem cell technology and bioceramics: From cell to gene engineering. J. Biomed. Mater. Res..

[67] Barrere F., van der Valk C.M., Meijer G., Dalmeijer R.A., de Groot K., Layrolle P. (2003). Osteointegration of biomimetic apatite coating applied onto dense and porous metal implants in femurs of goats. J. Biomed. Mater. Res. B.

[68] Li P. (2003). Biomimetic nano-apatite coating capable of promoting bone ingrowth. J. Biomed. Mater. Res. A.

[69] Yan W.Q., Nakamura T., Kawanabe K., Nishigochi S., Oka M., Kokubo T. (1997). Apatite layer-coated titanium for use as bone bonding implants. Biomaterials.

[70] Nagano M., Kitsugi T., Nakamura T., Kokubo T., Tanahashi M. (1996). Bone bonding ability of an apatite-coated polymer produced using a biomimetic method: A mechanical and histological study *in vivo*. J. Biomed. Mater. Res..

[71] Tanahashi M., Matsuda T. (1997). Surface functional group dependence on apatite formation on self-assembled monolayers in a simulated body fluid. J. Biomed. Mater. Res..

[72] Filmon R., Grizon F., Basle M.F., Chappaard D. (2002). Effects of negatively charged groups (carboxymethyl) on the calcification of poly(2-hydroxyethyl methacrylate). Biomaterials.

[73] Liu Y., Layrolle P., de Bruijn J., van Blitterswijk C., de Groot K. (2001). Biomimetic coprecipitation of calcium phosphate and bovine serum albumin on titanium alloy. J. Biomed. Mater. Res..

[74] Liu Y., Huse R.O., de Groot K., Buser D., Hunziker E.B. (2007). Delivery mode and efficacy of BMP-2 in association with implants. J. Dent. Res..

[75] Liu Y., Hunziker E.B., Layrolle P., van Blitterswijk C., Calvert P.D., de Groot K. (2003). Remineralization of demineralized albumin-calcium phosphate coatings. J. Biomed. Mater. Res. A.

[76] Wu G., Hunziker E.B., Zheng Y., Wismeijer D., Liu Y. (2011). Functionalization of deproteinized bovine bone with a coating-incorporated depot of BMP-2 renders the material efficiently osteoinductive and suppresses foreign-body reactivity. Bone.

[77] Wu G., Liu Y., Iizuka T., Hunziker E.B. (2010). Biomimetic coating of organic polymers with a protein-functionalized layer of calcium phosphate: The surface properties of the carrier influence neither the coating characteristics nor the incorporation mechanism or release kinetics of the protein. Tissue Eng. C.

[78] Barrere F., van B.C., de G.K., Layrolle P. (2002). Nucleation of biomimetic Ca-P coatings on ti6A14V from a SBF x 5 solution: Influence of magnesium. Biomaterials.

[79] Barrere F., van Blitterswijk C.A., de Groot K., Layrolle P. (2002). Influence of ionic strength and carbonate on the Ca-P coating formation from SBFx5 solution. Biomaterials.

[80] Barrere F., Layrolle P., van Blitterswijk C.A., de Groot K. (2001). Biomimetic coatings on titanium: A crystal growth study of octacalcium phosphate. J. Mater. Sci. Mater. Med..

[81] Liu Y., de Groot K., Hunziker E.B. (2005). BMP-2 liberated from biomimetic implant coatings induces and sustains direct ossification in an ectopic rat model. Bone.

[82] Wernike E., Hofstetter W., Liu Y., Wu G., Sebald H.J., Wismeijer D., Hunziker E.B., Siebenrock K.A., Klenke F.M. (2009). Long-term cell-mediated protein release from calcium phosphate ceramics. J. Biomed. Mater. Res. A.

[83] Liu Y., Enggist L., Kuffer A.F., Buser D., Hunziker E.B. (2007). The influence of BMP-2 and its mode of delivery on the osteoconductivity of implant surfaces during the early phase of osseointegration. Biomaterials.

[84] Wu G., Liu Y., Iizuka T., Hunziker E.B. (2010). The effect of a slow mode of BMP-2 delivery on the inflammatory response provoked by bone-defect-filling polymeric scaffolds. Biomaterials.

[85] Karageorgiou V., Kaplan D. (2005). Porosity of 3D biomaterial scaffolds and osteogenesis. Biomaterials.

[86] Liu Y., Hunziker E.B., Layrolle P., de Bruijn J.D., de Groot K. (2004). Bone morphogenetic protein 2 incorporated into biomimetic coatings retains its biological activity. Tissue Eng..

[87] Luong L.N., Hong S.I., Patel R.J., Outslay M.E., Kohn D.H. (2006). Spatial control of protein within biomimetically nucleated mineral. Biomaterials.

[88] Yu X., Qu H., Knecht D.A., Wei M. (2009). Incorporation of bovine serum albumin into biomimetic coatings on titanium with high loading efficacy and its release behavior. J. Mater. Sci. Mater. Med..

[89] Franceschi R.T. (2005). Biological approaches to bone regeneration by gene therapy. J. Dent. Res..

[90] Bonadio J., Smiley E., Patil P., Goldstein S. (1999). Localized, direct plasmid gene delivery *in vivo*: Prolonged therapy results in reproducible tissue regeneration. Nat. Med..

[91] Oakes D.A., Lieberman J.R. (2000). Osteoinductive applications of regional gene therapy: *Ex vivo* gene transfer. Clin. Orthop. Relat. Res..

[92] Anderson W.F. (1998). Human gene therapy. Nature.

[93] Evans C.H., Robbins P.D. (1995). Possible orthopaedic applications of gene therapy. J. Bone Jt. Surg. Am..

[94] Jenkins D.D., Yang G.P., Lorenz H.P., Longaker M.T., Sylvester K.G. (2003). Tissue engineering and regenerative medicine. Clin. Plast. Surg..

[95] Mahr J.A., Gooding L.R. (1999). Immune evasion by adenoviruses. Immunol. Rev..

[96] Noguchi P. (2003). Risks and benefits of gene therapy. N. Engl. J. Med..

[97] Wiethoff C.M., Middaugh C.R. (2003). Barriers to nonviral gene delivery. J. Pharm. Sci..

[98] Kolk A., Haczek C., Koch C., Vogt S., Kullmer M., Pautke C., Deppe H., Plank C. (2011). A strategy to establish a gene-activated matrix on titanium using gene vectors protected in a polylactide coating. Biomaterials.

[99] Jiang Q.H., Liu L., Shen J.W., Peel S., Yang G.L., Zhao S.F., He F.M. (2012). Influence of multilayer rhBMP-2 DNA coating on the proliferation and differentiation of MC3T3-E1 cells seeded on roughed titanium surface. J. Biomed. Mater. Res. A.

[100] Chen S., Yang J., Wang H., Chao Y., Zhang C., Shen J., Zhang P. (2013). Adenovirus encoding BMP-7 immobilized on titanium surface exhibits local delivery ability and regulates osteoblast differentiation *in vitro*. Arch. Oral Biol..

[101] McCarty D.M., Monahan P.E., Samulski R.J. (2001). Self-complementary recombinant adeno-associated virus (scAAV) vectors promote efficient transduction independently of DNA synthesis. Gene Ther..

[102] Dupont K.M., Boerckel J.D., Stevens H.Y., Diab T., Kolambkar Y.M., Takahata M., Schwarz E.M., Guldberg R.E. (2012). Synthetic scaffold coating with adeno-associated virus encoding BMP2 to promote endogenous bone repair. Cell Tissue Res..

[103] Ben Arav A., Pelled G., Zilberman Y., Kimelman-Bleich N., Gazit Z., Schwarz E.M., Gazit D. (2012). Adeno-associated virus-coated allografts: A novel approach for cranioplasty. J. Tissue Eng. Regen. Med..

